# The impact of the Affordable Care Act on patient coverage and access to care: perspectives from FQHC administrators in Arizona, California and Texas

**DOI:** 10.1186/s12913-021-06961-9

**Published:** 2021-09-06

**Authors:** Angelo Ercia

**Affiliations:** 1 Centre for Health Informatics, Division of Informatics, Imaging and Data Sciences, School of Health Sciences, Faculty of Biology, Medicine and Health, The University of Manchester, Jean McFarlane Building, Oxford Road, Manchester, M13 9PT UK; 2 Cievert, an Evergreen Life Company, Evergreen Business Centre, Clowes St, Manchester, M3 5NA UK

**Keywords:** Affordable care act, Federally Qualified Health Centers, Medicaid, Private insurance, Access to care, Health policy

## Abstract

**Background:**

The Affordable Care Act (ACA) enabled millions of people to gain coverage that was expected to improve access to healthcare services. However, it is unclear the extent of the policy’s impact on Federally Qualified Health Centers (FQHC) and the patients they served. This study sought to understand FQHC administrators’ views on the ACA’s impact on their patient population and organization. It specifically explores FQHC administrators’ perspective on 1) patients’ experience with gaining coverage 2) their ability to meet patients’ healthcare needs.

**Methods:**

Twenty-two semi-structured interviews were conducted with administrators from FQHCs in urban counties in 2 Medicaid-expanded states (Arizona and California) and 1 non-expanded state (Texas). An inductive thematic analysis approach was used to analyze the interview data.

**Results:**

All FQHC administrators reported uninsured patients were more likely to gain coverage from Medicaid than from private health insurance. Insured patients generally experienced an improvement in accessing healthcare services but depended on their plan’s covered services, FQHCs’ capacity to meet demand, and specialist providers’ willingness to accept their coverage type.

**Conclusion:**

Gaining coverage helped improved newly insured patients’ access to care, but limitations remained. Additional policies are required to better address the gaps in the depth of covered services in Medicaid and the most affordable PHI plans and capacity of providers to meet demand to ensure beneficiaries can fully access the health care services they need.

**Supplementary Information:**

The online version contains supplementary material available at 10.1186/s12913-021-06961-9.

## Background

The Patient Protection and Affordable Care Act (ACA) of 2010 was a comprehensive national health care reform aimed to expand health insurance coverage and improve access to care in the United States (US). The ACA enabled people to gain coverage by 1) expanding the publicly funded Medicaid program to cover adults with annual incomes up to 138% of the federal poverty level; 2) establishing the Health Insurance Marketplace for individuals and small businesses, allowing them to purchase private health insurance (PHI); and 3) enforcing an individual mandate that required eligible people to have federally approved health insurance coverage [1–4].

While the policy was passed in 2010, the provisions to expand coverage took effect in 2014 and their implementation varied from state to state. Some states did not expand the Medicaid program because of the Supreme Court’s 2012 ruling that made it an option [2]. As of 2020, 39 states, including the District of Columbia (D.C.), opted to expand Medicaid while 12 states decided against implementing the expansion [5]. Despite the inconsistent implementation of coverage expansion across the nation, the ACA enabled millions of uninsured people to gain coverage. An estimated 10.8 million low-income uninsured individuals enrolled in Medicaid in 2014 [6] and this increased to 12.2 million people by 2015 [2]. The Health and Human Services estimated that 11.7 million people in 2014 enrolled in a PHI plan [6, 7]. The National Health Interview Survey estimated the rate of uninsured people dropped to 9% by 2015 [8]. Several studies [9–11] have highlighted the different decline of uninsurance between Medicaid expanded and non-expanded states-- the former experiencing a larger decline in their uninsurance rates.

It was expected the ACA’s coverage expansion would improve access to care [12]. However, inconclusive evidence from several studies suggests it is unclear whether it has been realized, particularly among newly insured patients. For example, Shartzer et al. [7] suggest that access to care improved between 2013 and 2015 among nonelderly adults. Key informants (e.g., Medicaid and marketplace officials, assisters and advocates) interviewed in 4 Medicaid-expanded states (Colorado, Connecticut, Kentucky, and Washington) in 2016 believed Medicaid patients generally had good access to care, but acknowledged that limitations remained [13]. Wherry and Miller’s [14] findings suggest the evidence supporting improvements in access to care in Medicaid-expanded states was also inconsistent.

Furthermore, there is limited understanding of the ACA’s coverage expansion effect on newly insured low-income patients’ access to care served by Federally Qualified Health Centers (FQHCs). FQHCs are an integral part of the US’s safety net system as it provides primary care services to millions of vulnerable and underserved populations [10–12]. It has been estimated that FQHCs served one in 11 people in the nation [15]. The ACA’s coverage expansion was expected to enable many uninsured patients served by FQHCs to gain coverage from Medicaid and help them have better access to care. However, few studies have explored this topic. Findings have suggested that newly insured FQHC patients may have continued to experience limitations in accessing care. For example, some studies found that FQHCs in Medicaid-expanded states experienced an increase in their visit rates compared to non-expanded states [16, 17]. Angier et al. [18] also found FQHCs in five expanded states (California, Minnesota, Ohio, Oregon and Washington) experienced a 32% increase or 71 more visits per month among Medicaid patients. These findings suggest the immediate rise in demand may have challenged FQHCs to meet higher demand for care, particularly in sites that had limited capacity prior to the ACA taking effect. Some studies [13, 19] also have found that improvements in accessing care depended on primary and secondary care providers’ willingness to accept certain coverage type. Newly insured patients, particularly with Medicaid, could not always get care from their chosen provider, as it was not widely accepted [19, 20]. Therefore, this study aimed to gain insights from FQHC administrators in Medicaid expanded and non-expanded states on the impact introduced by the ACA on their patient population and organization. Specific objectives were to investigate 1) administrators’ views on patients’ experience with gaining coverage and 2) administrators’ views on their ability to meet patients’ healthcare needs.

## Methods

This study conducted semi-structured interviews with key informants that included executive directors and mid-level managers from selected FQHCs in urban counties of Arizona (AZ), California (CA), and Texas (TX). The study selected administrators from FQHCs in Medicaid-expanded states and a non-expanded state to understand their experiences with the ACA’s coverage expansion. California was selected because it expanded Medicaid and TX was selected because it did not expand the program. Arizona was included because it was a state that reluctantly expanded Medicaid. These three states were also selected because of similar characteristics of being Border States in the lower part of the US and continued increase in population growth [21]. Convenience sampling was used to select all FQHCs. AE initially identified FQHCs within a selected area comprised of urban counties in the three states from his pre-exiting professional network, as this provided a point of contact. A web search was then conducted to identify other FQHC sites in the area outside AE’s network. An FQHC was selected if they were classified as a Health Resources and Services Administration grantee and a community health center. At least one selected FQHC site had a large patient population (over 50,000) and a small patient population (under 50,000) in each selected area. This study used some data collected for AE’s PhD thesis [22] and received ethical approval from the University of Edinburgh School of Social and Political Science.

### Participants and recruitment

Key informants were selected to be interviewed if they held an executive director and mid-level manager position in the selected FQHCs. Executive directors were selected given their oversight of the strategic and financial management of their respective organizations [23]. They supported mid-level managers to oversee the impact of the ACA on their programs and patients. Mid-level managers were selected given their unique role of managing social structures and organizational strategic plans, while also managing day-to-day activities on their site [24]. The selected managers also supervised clinical services, patient outreach, and registration of health insurance plans, which were affected by the ACA. Convenience sampling was used to recruit participants through the primary author’s pre-existing professional networks, web searches, and social media. AE communicated with all the participants through email, which included inviting them to take part and scheduling the interview. The snowballing approach was also used to identify other participants suitable for the study. Participants that either declined or did not respond to the invitation were replaced by someone with a similar background in the organisation.

### Data collection

AE conducted all the interviews and took place during the selected timeframe of the study from July and September 2014. The majority of the interviews were conducted in the administrators’ office or in a meeting room within their FQHC. One interview was conducted on the telephone and another occurred in a public meeting space. Most of the interviews were completed individually. One interview was conducted with two participants from the same organization because of convenience. Participants completed a written consent form at the start of the interview and were aware that their participation was voluntary, with no compensation. The interview lasted for 60 minutes, conducted in English, and was guided by a topic guide (see Supplementary A). The design of the topic guide was primarily informed by the research question and current literature. As there is a gap in knowledge in the ACA’s direct impact on primary care providers, particularly among FQHCs’ patient population and organization, most of the questions in the guide aimed to explore these topics. The topic guide included questions that explored administrators’ perspectives on the impact of the ACA on their uninsured patients’ ability to gain coverage, their ability to meet patients’ healthcare needs, and challenges and opportunities with coverage expansion. The interviews were all audio recorded and then transcribed verbatim by the primary author.

### Data analysis

AE reviewed several transcripts and inductively coded the interviews to develop an initial coding framework guided by the research question. Several meetings took place with AE and two other members of the research team to discuss the suitability of the framework. Multiple meetings took place to discuss the coding process and the outcome of taking a constant comparative approach. NVivo 10 software was used to conduct this process. The coded data was then thematically analyzed [25] and presented to the research team to discuss emerging themes. The team met several times to discuss the themes as related to the research question until consensus was reached.

## Results

Ten FQHCs in two Medicaid expanded-states (AZ, CA) and one non-expanded state (TX) were selected to be part of this study. Four FQHCs were selected in AZ, 4 in CA, and 2 in TX. Twenty-two interviews were conducted and at least one executive director and one manager were interviewed in each site. There were instances that 2 executive directors and 2 managers were interviewed in some sites because of their availability. A total of 11 executive directors and 11 managers were interviewed (see Table 1). All FQHCs were in areas comprised of urban counties and offered similar comprehensive primary care services, dental care, mental health, health education, enabling and outreach services. All sites had a central site and multiple satellite sites. The patient volume served at all sites varied. At least one FQHC in each state served over 90,000 patients, and at least one FQHC served less than 50,000 patients.

**Table 1:** Interviews conducted

FQHC administrator location of interviews	Number of interviewees	Total
**Arizona**		**7**
Executive directors	3	
Managers	4	
**California**		**9**
Executive directors	4	
Managers	5	
**Texas**		**6**
Executive directors	4	
Managers	2	
**TOTAL**		**22**

### Administrator views on FQHC patients’ ability to gain coverage

All the interviewed FQHC administrators had positive views on expanding Medicaid to cover more low-income uninsured adults. Arizonan and Californian administrators were particularly positive about Medicaid expansion, as their state enacted the provision.

*“I think the biggest impact of the Affordable Care Act so far has been the dramatic increase in the number of patients that we see who has Medi-Cal [California’s Medicaid program].”* (CA Director 2)

Most administrators from AZ and CA estimated that the program’s expansion increased their sites’ newly insured Medicaid patients by 10 to 15%. However, directors and managers believed the increase was determined by the proportion of uninsured patients in their community that met the new eligibility criteria for Medicaid. Some FQHCs served communities with high uninsured populations that were eligible for Medicaid under the expanded eligibility criteria. Other FQHCs served communities with high immigrant and undocumented populations that were ineligible for Medicaid because of their immigration status. Arizona director 3 described their site as experiencing a 10% increase of newly insured Medicaid patients. However, they continued to serve a high proportion of uninsured patients who were ineligible for Medicaid because of their immigration status. Several other administrators in AZ, CA, and TX acknowledged that patients’ immigration status was a major barrier to gaining Medicaid. Therefore, FQHC administrators continued to depend on unrestricted locally funded programs to help subsidize the cost of care for these patients.

All Texan administrators believed their state’s decision to not expand Medicaid was a missed opportunity for their FQHC. They believed expanding the program would have enabled many of their low-income uninsured patients to gain coverage from Medicaid and reduce the proportion of patients without coverage they served. It would have also enabled their organization to generate more revenue that could help expand their capacity and resources to meet demand for care. While TX has yet to adopt Medicaid expansion, most directors believed their state would expand the program eventually in some form that best meets the needs of their population.

*“Texas has said that they are not going to expand Medicaid, but I don’t think that means they are not going to do anything, right? They are going to do something, they just gotta figure out what works for Texas … it just probably won’t look like how the Feds (US Federal government) originally designed it. [It] will look like something Texas designed.”* (TX Director 2)

All Arizonan and Californian administrators acknowledged their uptake of newly insured patients with PHI was minimal. Texan FQHCs experienced a higher uptake of patients with PHI compared to AZ and CA FQHCs, but administrators did not view this as significant. Two Texan directors from the same FQHC stated 1% of their patients had PHI prior to the ACA and only increased to 5% after the enactment of the ACA.

All administrators from the three states acknowledged PHI remained unaffordable for many of their low-income patients. Patients with annual incomes slightly surpassing the Medicaid income eligibility risked not being able to afford the monthly premium and out-of-pocket expenses of a marketplace PHI plan even with Federal government subsidies. Most of the managers also believed the patients that could purchase a marketplace PHI plan would experience financial hardship in maintaining their plan.

*“I think most our patients are making the decision whether they want to buy groceries or go to the doctor. And they don’t have the money for even an inexpensive insurance program … it’s like these people not only live pay check to pay check … They were already coming to see us with no money.”* (TX Manager 1)

All administrators from the three states believed low-income FQHC patients that purchased a PHI plan selected the most affordable plan (known as the bronze plan). These plans had limited provider networks and high out-of-pocket expenses, thus limiting beneficiaries’ access to care. AZ Director 2 stated, *“generally the well visits are covered but if [they] end up needing acute care [their] insurance may not pay much at all*. *.. maybe [their] deductible is $2,500 before [their] insurance really kicks in”.* Several directors and managers were also concerned that their low-income patients with PHI were underinsured, a problem that seemed to grow under the ACA. TX Manager 2 believed these plans give their patients a sense of “*falsehood of being insured when really, they [can’t afford] insurance”.*

### Challenges of newly insured patients to access primary care services from FQHCs

All the administrators in the three states viewed the Medicaid program as an effective form of coverage for their low-income patients. It enabled beneficiaries to access preventative and primary care services with no, or limited, out-of-pocket expenses. However, Arizonan and Californian administrators were concerned that fewer non-FQHC primary care providers (PCPs) (e.g., private providers) accepted new Medicaid patients to establish care with them. Therefore, it restricted new Medicaid patients’ choice of PCPs and, to an extent, caused them to rely on establishing care with an FQHC. This caused FQHCs to see an increase in serving more insured patients. Most managers in AZ and CA believed the rapid gains of newly insured patients, particularly with Medicaid, further increased their demand and affected access to care. CA Manager 2 believed, *“[taking] a large group of people who formerly didn’t have any health insurance coverage and [are given] coverage overnight. .. these people have all these pent up health care needs. .. now they are flooding the system, they have an ‘[insurance] card’ so they think they should get everything in today and rightfully so”*. Managers from other FQHCs also believed newly insured patients had many neglected health conditions that were not treated when they were uninsured. Many patients required multiple treatments and referrals. According to CA manager 4, “*it’s not like [a patient] comes in here today and get a flu shot. .. [they] get so many referrals, [they need] so much help”*.

The rapid rise of serving newly insured Medicaid patients with co-morbidities that needed multiple treatment caused many patients in AZ and CA to experience longer waits for an appointment. CA Manager 1 stated “*someone might attempt to schedule an appointment to establish care, and for those type of appointments it can take as long as three months*”. Directors and managers from TX did not associate the increase in demand they continued to experience after the ACA took effect because of the limited impact of coverage expansion. They believed local events such as recent rises in migration into their city more likely contributed to the increased number of patients seeking care from them.

All administrators from the three states believed they would continue to struggle to meet demand unless they expanded their capacity, something that had proven difficult because of limited financial resources and workforce shortages. This limited capacity stopped some FQHCs accepting new patients, which directly affected patients’ ability to establish care with them. TX Director 3 acknowledged limited capacity meant, “*the [staff] have to explain to patients that [they] are not accepting new patients. They give them the number for the two other FQHCs [that] are accepting new patients and until we get the new site, that’s the best we can do”*.

### Challenges of newly insured patients’ access to secondary care services

Most of the administrators discussed struggling to refer their newly insured patients to secondary care because of the large volume of need. CA Manager 4, in agreement with the perspectives of the other administrators in AZ and TX, stated:

*“When they come to us (patients), they [need] four or five referrals. .. they need to see a cardiologist, they need to see a gastroenterologist. They have so much going on and I don’t think we were expecting that.”*

This study found that some newly insured patients’ coverage plan restricted their ability to access secondary care. All administrators from the three states acknowledged that referring Medicaid patients to specialists pre-ACA was a challenge, as not all specialists accepted the coverage. Most Arizonan and Californian administrators also believed specialists became more selective about the type of coverage they accepted after the ACA took effect. Some administrators described that there were only a handful of secondary care providers in their region willing to accept patients with Medicaid. Some directors aimed to form partnerships with local specialists to serve their patients, but this did not guarantee patients access to timely secondary care. The majority of administrators in the three states also struggled to refer patients with the most affordable PHI plan (bronze plan) to specialists, as these plans had a very narrow network of specialist providers willing to accept the coverage and high out-of-pocket expenses. AZ Director 2 stated,

“*We worry that they (patients) are going to need specialty care and it’s not going to be available. .. the network is going to be so narrow that it’ll be challenging to find them specialty providers”*.

Constant shortages of specialists across regions further challenged patients’ ability to access secondary care services because of long waiting times. AZ Director 1 stated, *“If you need a rheumatology referral, we are talking [a] three or four months [wait]”*. A Californian manager acknowledged that many of their local specialists were also reaching maximum capacity. The manager described that there was a 6 month waiting period for physical therapy referrals in the county general hospital. Sometimes patients were referred to specialists outside their county because of lack of appointment availability. This was a barrier for many FQHC patients, as it required them to take time off from work and potentially travel long distances.

## Discussion

The ACA’s multi-faceted approach to expanding coverage enabled millions of people in the US to gain coverage in a short period of time [26, 27]. However, this study found that administrators believed Medicaid expansion was the key element in providing coverage to low-income uninsured patients served by FQHCs in urban counties of AZ and CA. The absence of Medicaid expansion in TX placed many low-income patients of FQHCs at risk of remaining uninsured as marketplace PHI plans remained unaffordable. Moreover, all the administrators believed Medicaid was the most appropriate form of coverage for their low-income patients because of its comprehensive coverage for primary care services and limited or no out-of-pocket expenses. Health care professionals from small private practices, FQHCs, free/low-cost clinics, and hospital-based practices in other states such as Michigan also had this view [28].

The findings of this study and others [13, 28] suggests newly insured Medicaid patients experienced an improvement in accessing care under the ACA. However, this study highlights exceptions as some newly insured continued to experience limitations with accessing care and, sometimes, contributed to the growing problem of underinsurance. For example, some private PCPs in AZ and CA did not accept newly insured Medicaid patients to establish care with them. Some newly insured Medicaid patients, therefore, had limited choice of PCPs and, to an extent, relied on establishing care with FQHCs when no other providers would accept them. This is supported in Boccuti et al. [29] analysis of a nationwide survey of primary care providers, as they found that only 45% of non-paediatric PCPs accepted new Medicaid patients- a proportion much lower compared to accepting patients with Medicare (72%) or private insurance (80%). Other studies [4, 30–32] also found that compared to privately insured patients, more Medicaid patients struggled to get appointments with primary care providers. A contrasting viewpoint comes from Polsky et al. [26] which found the ACA’s temporary introduction of higher payment rates for PCPs serving Medicaid patients improved patients’ ability to get an appointment in 10 states. It was unclear, however, whether PCPs would continue accepting new Medicaid patients and offer appointments after the temporary payment increase ended.

Besides the challenges with establishing care with private PCPs, administrators believed newly insured patients with Medicaid and the most affordable PHI plan experienced difficulties in accessing secondary care services. Most administrators in AZ, CA, and TX acknowledged the challenge of referring their Medicaid patients to secondary care, both before and after the enactment of the ACA. However, they observed that newly insured patients with Medicaid or marketplace PHI plan continue to struggle to access secondary care services given the very narrow network of specialists willing to accept their coverage. Out-of-pocket expenses of affordable PHI plan were also high and unaffordable. These findings have been found by other studies [13, 24, 33–35] and suggest that gaining insurance did not necessarily protect newly insured FQHC patients from becoming underinsured.

Although the depth of covered services of Medicaid and certain PHI plans influenced newly insured patients’ ability to access care, the study findings also suggest the capacity of healthcare providers to provide care was a significant factor. All FQHC administrators in the three states acknowledged their organization struggled to meet demand because of their limited capacity even before the ACA took effect. Arizonan and Californian administrators believed coverage expansion further exacerbated this problem, as they served more newly insured Medicaid patients that sought care for multiple untreated health conditions. Many newly insured patients had comorbidities that required extensive treatment and referrals to secondary care services. The analysis of the Community Health Applied Research Network database that composed of 17 FQHCs in nine states also found demand increased under the ACA because of serving new young patients with chronic physical and/or mental health conditions requiring multiple primary and secondary treatments [27]. The high demand prior to and after the implementation of the ACA with constant limited capacity, thus made it more difficult for these primary care providers to meet the needs of newly insured patients.

The limited capacity of secondary care providers affected their ability to meet demand for care, particularly in regions with high specialist provider shortages. This was not unique to AZ, CA, and TX, as Goold et al. [28] found specialist shortages occurred in rural and urban areas across Michigan. Nakamura et al. [36] also suggest that access to specialty care depended on the availability of specialists in the region. Administers in this study believed it caused patients to experience longer waiting period for appointments, travel farther distances to receive care, or could not access secondary care altogether. As a result, it reduced FQHCs’ ability to effectively care for their patients and contributed to the rise in patients developing unmet medical needs that required additional services, including emergency services [37].

### Policy implication and limitation of the study

This study adds to the literature insights from FQHC administrators on their experience with the ACA’s impact on their patient population and organization. It expands knowledge in understanding how the design of the ACA in expanding coverage and improving access to care translated into practice among FQHCs and the patients they served.

A key finding in the study identified the covered services of Medicaid and certain private insurance plans (e.g., bronze plan) were limited and not all providers accepted them. This caused patients with the coverage type to continue experiencing barriers in accessing primary and secondary care services, particularly in areas that had few providers. It underscores the need for additional policies in these plans to be widely accepted as to prevent patients from having health insurance coverage but unable to establish care with a provider or access to affordable health care services. This issue is a nationwide problem, as scholars and policymakers in other states have identified the need to address it. The state of Michigan considered setting up local incentives for providers to encourage acceptance of all forms of coverage [12]. Colorado policy makers considered increasing reimbursement rates for providers that accepted Medicaid [13]. Kentucky and Washington policymakers considered allowing more primary and secondary care providers to join the provider network that offers services to Medicaid patients [13]. Improving the network of secondary care providers that accept Medicaid and all forms of marketplace PHI plan is also imperative to minimize unmet needs and exacerbating patients’ health conditions that cannot be treated from primary care alone. While local and state level policies may be an effective initial step in addressing this problem, a comprehensive national approach could better address this issue that could minimize different practices across states.

Newly insured patients’ ability to access primary healthcare services relied on FQHCs’ capacity. Our findings suggest that many of the FQHCs were challenged to effectively meet the needs of their patients when they reach their maximum capacity. Expanding capacity to meet higher demand was also a struggle for most FQHCs as this was a lengthy process that could be affected by external factors such as availability of funding and access to the healthcare workforce. This was not a unique problem in this study as [13] found that FQHCs in Colorado, Connecticut, Kentucky, and Washington also had the same issue. Furthermore, Artiga et al. [13] found hiring more primary care providers became more challenging under the ACA because of intense competition among all healthcare providers to hire more of them. FQHCs in Colorado also struggled to recruit and retain clinical staff because of their inability to provide competitive salaries that the private sectors could offer [13]. Policy makers need to consider a strategy in which FQHC providers can hire and retain more healthcare professionals to expand their capacity without the financial competition from private providers. Otherwise, FQHCs will continue to struggle to recruit for more personnel and will remain in a constant state of trying to keep up with demand.

This study has several limitations that should be considered. First, the interviews were conducted with FQHC administrators working in urban counties in AZ, CA, and TX. Their experiences and perspectives differ from those of FQHCs in other parts of the state and country, particularly in rural areas. Second, the perspectives of the administrators reflected the beginning of the ACA’s implementation of coverage expansion. California expanded Medicaid in October 2013 and Arizona expanded in January 2014, thus the views of administrators reflected their experiences during the early stages of the policy implementation and several months thereafter. The study also focused on understanding the views of executive directors and mid-level managers. While this provided unique insights into the impact of the ACA’s coverage expansion on FQHCs, these insights do not reflect the experiences of patients and wider staff members—particularly clinicians. Third, these states are along the border of Mexico, thus exposing them to unique factors caused by migration and immigration policies. Many of the administrators in AZ, CA, and TX acknowledged the immediate impact of state- and federal-level immigration policies. Remaining well-informed of current immigration policies was important, given their impact on the organization and patient population. Last, Texas was the only state selected that did not expand Medicaid in the sample. Therefore, the perspective of administrators from the state may be unique and does not reflect other non-expanded states’ experiences to be used exclusively as a comparison to Medicaid-expanded states.

## Conclusion

This study presents FQHC administrators’ views on the ACA’s impact on their patient population and organization. The findings suggest the ACA’s coverage expansion provided the opportunity for uninsured low-income FQHC patients to gain coverage. However, uninsured FQHC patients living in Medicaid expanded states (AZ and CA) were more likely to gain coverage than those living in the non-expanded state (TX). PHI from the marketplace remained unaffordable for most uninsured FQHC patients. While gaining coverage from Medicaid and the most affordable PHI plan enabled newly insured patients to experience an improvement in accessing care, gaps remained in the depth of covered services and willingness of all providers to accept them. Additional policies are needed to expand covered services of these coverage types and extend capacities of FQHCs to better meet higher demand for care.

## Supplementary Information

**Additional file 1.**

## Data Availability

The dataset analysed in this study are not publicly available due to potentially identifiable information about the key informants based on their full transcripts. De-identified data can be requested from the corresponding author upon reasonable request.

## Abbreviations

ACA: Affordable Care Act
AZ: Arizona
CA: California
FQHC: Federally Qualified Health Centers
PHI: Private health insurance
PCPs: Primary care providers
TX: Texas
