# Supplementary material for: The impact of the Affordable Care Act on patient coverage and access to care: perspectives from FQHC administrators in Arizona, California and Texas
Source: BMC Health Serv Res. 2021 Sep 6;21:920. doi: 10.1186/s12913-021-06961-9 (PMC8420058; doi:10.1186/s12913-021-06961-9)
Supplement: Supplementary file 1 — Additional file 1. [file 12913_2021_6961_MOESM1_ESM.docx]

**Supplementary A: Interview guide**

| **Investigating the impact of the Patient Protection & Affordable Care Act (ACA) on Federally Qualified Health Clinics**  **Interview Questions** |
| --- |

| Introduction  My name is __________ and I am the interviewer for you today.  Thank you for agreeing to participate with this interview. As mentioned from the participant information sheet, the purpose of the interview is to learn from your knowledge and experience of the impact that the ACA has made on your clinic’s ability to meet its mission of serving underserved population. The aim of the interview is to learn from you the following:   - Overall impact of the ACA on your clinic for the last 5 years and other significant events that influenced your clinic’s ability to serve underserved communities with a focus on low-income nonelderly adults. - Challenges experience by your clinic to meeting your mission under the ACA. - Opportunities gained by your clinic due to the implementation of the ACA that has helped meeting your mission. - Strategies that your clinic has adapted to help meet your clinic’s mission under the ACA.     As a participant of this interview, you will contribute to understanding the effects of the ACA on FQHCs ability to provide care to its target population. The interview will take an estimated 60 minutes and at any point you may end to stop the interview as you wish. I will also be recording the interview so I can capture all the information you share. All the information from this interview will be confidential and will be securely stored. |
| --- |

1. Can you please describe the mission of your clinic in one sentence?
2. What were the changes your clinic experiences the last 5-10 years that have affected your ability to meet your mission?

- Affected your ability to serve low-income nonelderly adults?

1. How did the Affordable Care Act (ACA) affect your clinic’s ability to meet its mission of serving underserved population specifically the low-income non-elderly adults since 2010?

| (Supporting questions)  Did you get the sense that the expansion of health insurance coverage affected your clinic’s ability to meet your mission and serve low-income nonelderly adults?  Did you get the sense that the increase of funding to FQHCs from the federal government impacted your clinic’s ability to meet your mission and serve low-income nonelderly adults? |
| --- |

1. Has your clinic experience any challenges meeting its mission and serving low-income nonelderly adult due to the implementation of the ACA since 2010?

| (Supporting questions)  Has your clinic experience any challenges with serving low-income nonelderly adults as a result of the expansion of health insurance coverage?  Has your clinic experience any challenges with serving low-income nonelderly adults as a result of the increase in funding from the federal government?  Has your clinic experience any challenges serving uninsured, underinsured and undocumented adults under the ACA? |
| --- |

1. How did the challenges you identified affect your ability to meet the clinic’s mission and continue serving low-income nonelderly adult?

| (Supporting question)  How did the challenges you identified affect your clinic’s ability to serve: Uninsured, Underinsured, and Undocumented migrants seeking care in your clinic? |
| --- |

1. What were the strategies your clinic implemented as a result of the challenges your clinic face under the ACA to continue meeting the mission and serve low-income nonelderly adults?

| (Supporting question)  What were the strategies your clinic implemented as a result of challenges that affected your abilities to serve: Uninsured, Underinsured, and Undocumented migrant adults seeking care in your clinic? |
| --- |

1. Has your clinic experienced any opportunities under the ACA that has helped your clinic to meet its mission and continue serving low-income nonelderly adults since 2010?

| (Supporting questions)  What were the opportunities your clinic gained to serve low-income nonelderly adults as a result of the expansion of health insurance coverage?  What were the opportunities your clinic gained to serve low-income nonelderly adults as a result of the increase of funding from the federal government?  What were the opportunities your clinic gained to serve uninsured, underinsured and undocumented patients? |
| --- |

1. How did the opportunities your clinic gained affect your ability to continue serving low-income nonelderly adults?

| (Supporting question)  How did the opportunities your clinic gained affect your ability to serve: Uninsured, Underinsured, and Undocumented migrants seeking care in your clinic? |
| --- |

1. What are the strategies your clinic implemented as a result of the opportunities your clinic gained to continue serving low-income nonelderly adults?

| (Supporting question)  What were the strategies your clinic implemented as a result of the challenges that affected your abilities to serve: Uninsured, Underinsured, and Undocumented migrants seeking care in your clinic? |
| --- |

1. Is there anything else you would like to add that we did not discuss as a result of the implementation of the ACA and its effect on your clinic’s ability to continue serving low-income nonelderly adults?

| **Closing/ Debrief**  Thank you for participating with this interview. If you have any questions in the future about the project, you are welcome to contact me. In addition, if you would like to receive a summary of findings of the interviews, you may also use the contact information to request a copy of it. |
| --- |
